# Men’s perception of information and psychological distress in the diagnostic phase of prostate cancer: a comparative mixed methods study

**DOI:** 10.1186/s12912-022-01047-1

**Published:** 2022-09-30

**Authors:** Maja Elisabeth Juul Søndergaard, Kirsten Lode, Sissel Eikeland Husebø, Ingvild Dalen, Svein Reidar Kjosavik

**Affiliations:** 1 Department of Surgery, Stavanger University Hospital, Postboks 8100, 4068, Stavanger, Norway; 2 Research Group of Nursing and Healthcare Sciences, Stavanger University Hospital, Postboks 8100, 4068, Stavanger, Norway; 3 Faculty of Health Sciences, University of Stavanger, Kjell Arholms Hus, postboks 8600, 4036, Stavanger, Norway; 4 Department of Research, Section of Biostatistics, Stavanger University Hospital, Postboks 8100, 4068 Stavanger, Norway; 5 The General Practice and Care Coordination Research Group, Stavanger University Hospital, Postboks 8100, 4068, Stavanger, Norway

**Keywords:** Diagnostic phase, Distress, Information, Patient experience, Prostate cancer

## Abstract

**Background:**

Previous studies indicate that men experience frustration and uncertainty when confronted with an elevated prostate specific antigen (PSA) test and during further diagnostics for prostate cancer. The novel Stockholm3 test is an algorithm-based test that combines plasma protein biomarkers, genetic markers and clinical variables in predicting the risk of PCa. The test was introduced in a western part of Norway as a new tool for detecting prostate cancer. This study aimed to explore and compare men’s perception of information and possible experience of distress between a PSA group and a Stockholm3 group during the diagnostic phase of prostate cancer.

**Methods:**

This study is a part of the trailing research evaluating the impact of the change from PSA to Stockholm3. It is a multicenter study using a comparative mixed method design. Data were collected in a PSA group (*n* = 130) and a Stockholm3 group (*n* = 120) between 2017 and 2019. Quantitative data were collected using questionnaires and qualitative data were collected using semi-structured interviews (*n* = 20). The quantitative and qualitative data were analysed and compared separately and then merged in a side-by-side discussion. The study adheres to the GRAMMS guidelines for reporting mixed-methods research.

**Results:**

Compared with the PSA group, men in the Stockholm3 group reported that the information from the general practitioners was better. Similarly, men in the Stockholm3 group were more likely to indicate that they had received sufficient information regarding how examinations would be conducted. No differences were found between the groups regarding waiting time and distress. Three themes emerged from the qualitative analysis of the two groups: “Information affects the experience of comprehension”, “Stepping into the world of the healthcare system”, and “Periodically feelings of distress”.

**Conclusion:**

The Stockholm3 test may facilitate the provision of information to patients. However, some patients in both groups experienced distress and would benefit from more information and additional support from healthcare professionals. Routines that ensure sufficient information from the interdisciplinary healthcare team should be of priority during the diagnostic phase of prostate cancer in order to provide patients with predictability and to avoid unnecessary distress.

**Supplementary Information:**

The online version contains supplementary material available at 10.1186/s12912-022-01047-1.

## Introduction

Globally, prostate cancer (PCa) is the second most common cancer in men and accounts for about 15% of all cancer in men [1]. A patient’s referral to specialized health care for diagnosing PCa is usually based on an elevated prostate-specific-antigen (PSA) test and/or a digital rectal examination. Using PSA for screening has been evaluated by long-term randomized controlled trials and overdiagnosis has been estimated to occur in 21% to 50% of PCas detected [2]. International and national guidelines strongly recommend adequate information on potential risks and benefits to men before they undergo PSA testing [3, 4]. The health authorities in Norway do not recommend PSA screening for the general male population and the health services has a responsibility to limit overdiagnosis and unnecessary treatment. PSA tests are recommended for men with genetic predispositions, symptoms and/or palpation findings and only after sufficient information [5]. However, there is widespread unsystematic testing of men with PSA in Norway [6].

**Fig. 1:**
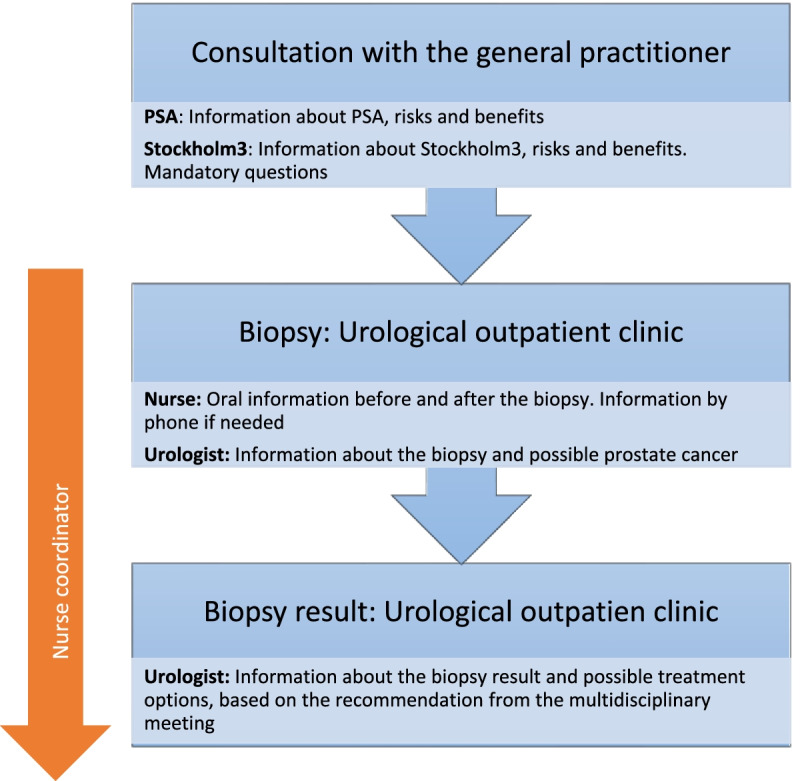
Flowchart of information points during diagnostic evaluation for prostate cancer

Previous studies suggest that men experience frustration and uncertainty regarding the limitations of the test and the further diagnostic process when confronted with an elevated PSA [7, 8]. The diagnostic phase of PCa refers to the period that consist of initial blood tests, clinical examination, possible prostate imaging and biopsies, as defined by the Norwegian health authorities [9]. According to the Essential Requirements for Quality Cancer Care (ERQCC), the diagnostic evaluation should be organized as an interdisciplinary collaboration in a standardized patient cancer pathway (SCP) in order to secure good patient-centred care. As a part of an interdisciplinary team, nurses have versatile and pivotal functions throughout the SCP, including being a key contact for patients and providing information, care, and support [10]. However, recent studies have found that men in the SCP of PCa require personalized information and that some men are in need of additional psychological support beyond the scope of the SCP [11, 12] (Fig. 1).

## Background

The interdisciplinary project “From PSA to Stockholm3” aims to improve the accuracy of PCa diagnostics, i.e. improve the diagnosis of clinically significant cancer while reducing overdiagnosis. The purpose of implementation was that all general practitioners (GPs) in the catchment area were recommended to change from PSA to Stockholm3 when conducting a risk-stratification for PCa [13]. The Stockholm3 test is an algorithm developed at the Karolinska Institute in Sweden. Based on blood tests (including PSA) and some clinical information, it aims to increase both sensitivity and specificity compared with the PSA, and thereby reduce the number of biopsies required without compromising the ability to diagnose clinically significant PCa (Gleason score of at least 7) [13–15]. The Stockholm3 test contains mandatory preliminary questions about three issues that should be discussed with the patient prior to the test, These questions are a part of the algorithm that determines the risk score and pertain to family history of PCa, use of medication related to the prostate, and any previous prostate biopsies [13].

Previous research may imply that PSA testing can be initiated by GPs and without patients being aware that PSA has been ordered along with other blood tests [16, 17]. Furthermore, the monitoring practices of patients with a raised PSA may leave uncertainty about further stages of the process [16]. Although men may be aware of the PSA test, insufficient knowledge about what the test entails has been reported [7, 18, 19]. For example, men were often unfamiliar with the limitations of the test (20). Patients have experienced lack of information during the trajectory of PCa, and the process of diagnostics and treatment was perceived as long and complex [12]. Furthermore, inadequate information can cause distress in patients with suspected PCa [7, 20]. Lower levels of knowledge about PCa and treatment options has shown to be associated with anxiety prior to diagnosis [21]. However, the patient’s perception of information received and possible distress during the diagnostic phase of PCa have not yet been thoroughly explored [19, 22]. Distress has been defined as “a multifactorial unpleasant experience of a psychological, social, spiritual, and/or physical nature that may interfere with the ability to cope effectively with cancer, its physical symptoms, and its treatment” [23]. Distress ranges from common feelings, such as sadness and fear, to depression, anxiety, and panic [23]. Previous research shows mixed findings concerning distress during the diagnostic phase of PCa. Some studies have found a low incidence of clinically significant anxiety in men with an elevated PSA, and that only a small group of men evaluated for PCa had significant psychological distress [24, 25]. In contrast, a review reported that 30–40% of men with suspected PCa reported that anxiety affected their day-to-day life [26]. Distress has been reported in 49% of men after attending for a prostate biopsy [27]. Different scales, cut-off scores, cultures, and times of data collection may explain some of the existing discrepancies in the research examining distress in the diagnostic phase of PCa [24]. However, it also reveals a need for a more comprehensive understanding of patients’ perception of the provided information and the possible experience of distress in the diagnostic phase of PCa. Comparing and synthesizing quantitative and qualitative data may add an important contribution to the existing literature [28]. Furthermore, the implementation of the Stockholm3 test may lead to changes in how patients experience the diagnostic evaluation for PCa. It may have consequences for how healthcare providers should approach and care for this group of patients.

The current study is a sub-study of the larger project “From PSA to Stockholm3”, and is characterized as trailing research because the research team took on a passive role regarding the implementation of the Stockholm3 test and its diagnostic performance. Instead, the research team engaged in the evaluation process of the participant’s experiences, which is the focal point of this study. This involved a scientific approach and a critical distance to actual action, in order to intercept changes that the Stockholm3 test may entail for patients regarding information and potential distress [29].

To our knowledge, there are no studies to date that have explored the patient perspective regarding the Stockholm3 test. Therefore, the aim of this study was to explore and compare men’s perception of information and possible experience of distress between a PSA group and a Stockholm3 group during the diagnostic phase of PCa.

## Methods

### Design

**Fig. 2:**
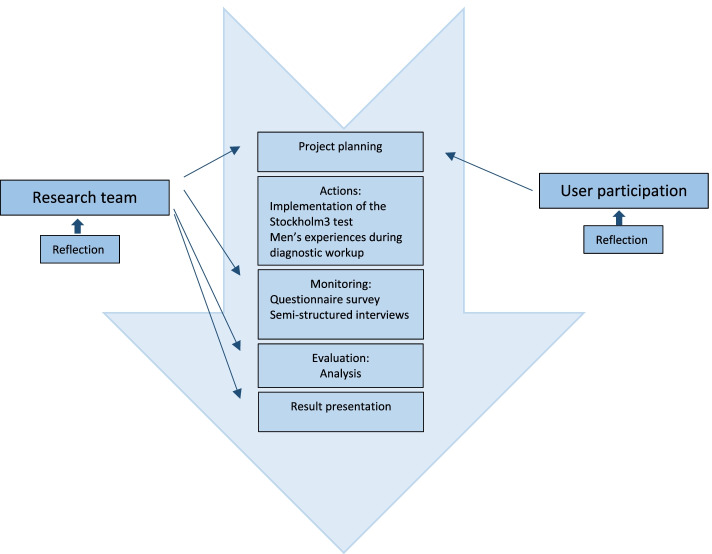
Flowchart of the study process using a trailing research approach

In this study, we used a trailing research design as an approach to study changes in real time without the research team acting as an agent of change. The objective was to generate insight about the change initiated by the implementation of the Stockholm3 test, by following academic procedures to analyze the process and to produce new knowledge while balancing with care to the practical context [30]. The Design implied a constant attention to our role as a research team, which was discussed throughout the entire process [31] (Fig. 2).

**Fig. 3:**
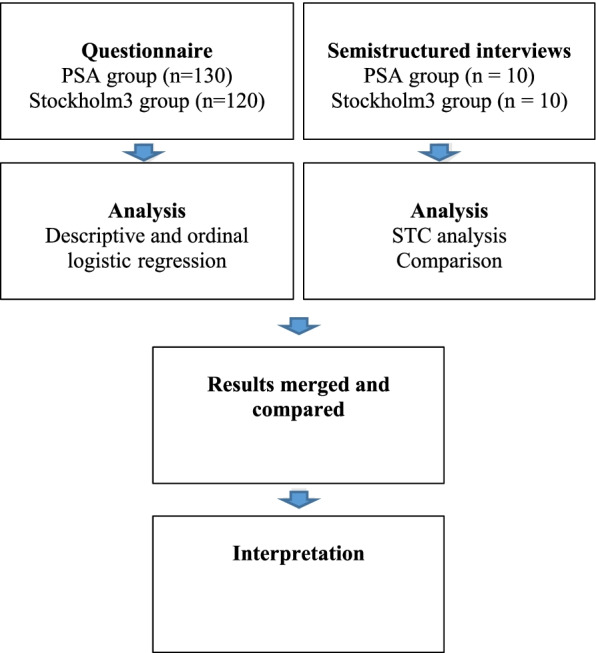
A Convergent mixed methods design, presenting data collection and analysis process

The two participant groups underwent similar diagnostic evaluation at the urological outpatient clinics, apart from being assigned an initial diagnostic test using either PSA or Stockholm3 procedures. There was no other special attention given to the Stockholm3 group. A comparative study using a convergent mixed method design was employed (Fig. 3).

This design means that parallel strands of qualitative and quantitative data that are analysed individually and then brought together during interpretation [28]. The comparison between the quantitative data of each group and the comparison between the qualitative data for the two groups constitutes an intermediate step that extended the core design to a more advanced design. After conducting the intermediate analysis, the quantitative and qualitative results were merged and interpreted in adherence with the core design [32]. The qualitative and quantitative methods were given equal priority. The study design was considered appropriate because existing knowledge is limited, and our aim was to enable a comparison of quantitative and qualitative results to produce a more detailed and complete understanding of the topic [28]. The Good Reporting of A Mixed Methods Study (GRAMMS) checklist was applied to enhance validity in the study [33] (see Additional file 1).

### Setting and sample

The study was conducted in the western part of Norway in three different settings. According to the original protocol, all data was supposed to be collected from a single clinic (Clinic I); however, the Stockholm3 test was implemented in Clinic I before the PSA data collection was completed, resulting in two more clinics being added (Clinic II and Clinic III). The three urological outpatient clinics were selected because they belonged to the same health trust and had similar procedures and routines during the diagnostic phase of PCa in accordance with the national SCP for PCa. However, Clinic I and Clinic II were affiliated with university hospitals in urban settings while Clinic III was affiliated with a hospital in a less urban setting. At Clinic I, both PSA and Stockholm3 data were collected, while at Clinic II and III only PSA data were collected because the Stockholm3 test was only implemented in Clinic I. Due to the geographical proximity of the research team, the individual semi-structured interviews were conducted at Clinic I. The interviews took place in suitable private rooms at the outpatient clinic, at an affiliated satellite clinic, or in a conference room at the hospital.

At all three clinics, patients were invited to participate in the study after they had received antibiotics and oral information and were awaiting the prostate biopsy at the clinics. A nurse or a study nurse who provided oral information about the biopsy invited patients to participate in the study. A convenience sample, based on accessibility and that met the inclusion criteria [34]. Inclusion criteria were: patients referred based on an elevated PSA test or an elevated Stockholm3 test, over 18 years of age, no prior diagnosis of PCa, and able to provide informed consent. Patients with cognitive impairment were excluded. Participants completed the questionnaire at one of the three urological outpatient clinics after receiving antibiotic and while they were awaiting their biopsy. Participants were also given the option of completing the questionnaire at home, and received a stamped envelope to return later. When patients agreed to participate in the questionnaire survey in Clinic I, the first author informed potential participants about the individual follow-up interview. A purposive sampling strategy was used in the individual interviews to ensure a variation in age and PSA or Stockholm3 value at the time of referral [35]. From the patients who completed the questionnaire at Clinic I, 10 patients with an elevated PSA test (PSA group) and 12 patients with an elevated Stockholm3 test (Stockholm3 group) were invited to participate in semi-structured interviews. The first author contacted the 22 patients that had been willing to participate in the interview by phone after they had received the biopsy result. Two patients from the Stockholm3 group declined to participate, one because of severe PCa, the other due to lack of time. Finally, 20 patients agreed to participate in the semi-structured interviews, distributed with 10 men in each group. The interviews were scheduled and conducted during the two weeks following their biopsy result.

### Data collection

Data were collected between September 2017 and November 2019. The recruitment of eligible participants for the survey was performed by different nurses at the three clinics. The first author assisted in the recruitment and data collection at Clinic I. All semi-structured interviews were conducted by the first author.

### Quantitative measurement

#### Demographics

Demographic information such as patients’ age, the number of people in their household, educational level, and occupation was gathered at the beginning of the survey.

#### Patient experience items

Four items addressing patient experiences with the diagnostic evaluation of cancer were selected from a national survey previously conducted on the general population and cancer patients in Norway [36]. The national survey aimed to explore different conditions and challenges in the healthcare services for cancer patients. The items used were: 1. “Did you find that your GP gave you satisfactory information about what was going to happen related to the diagnostic evaluation of possible prostate cancer?”, 2.“Did you find the waiting time from hospital referral until first attendance acceptable?”, 3.“Did you find that the referring doctor/GP and the hospital worked well together?” and, 4.“Were you told what you thought was necessary regarding how examinations would be conducted?” Participants responded on a five-point Likert scale (1 representing “not at all” and 5 representing “to a very large extent”) with an additional option of “not relevant.” A higher score indicated a more satisfying experience for each variable [36].

#### The hospital anxiety and depression scale

The Hospital Anxiety and Depression Scale (HADS) was developed as a screening instrument for assessing the likelihood of anxiety disorders and depression among patients in non-psychiatric hospital clinics. The scale consists of two subscales that each produce a score of 0–21. One scale assesses anxiety (HADS-A) while the other assesses depression (HADS-D). Together, the two subscales constitute HADS-T with a score from 0–42 [37]. The most optimal balance between sensitivity and specificity as a screening instrument for the two subscales was previously found to be a cut-off score of 8 + for patients with possible anxiety or depression [38]. Therefore, a cut-off score of ≥ 8 was used in the present study. HADS’ ability to identify possible distress is well described in the literature [39]. The Norwegian version of the HADS has been validated by Leiknes et al. [39].

### Qualitative interviews

An interview guide was developed by the research team to obtain an in-depth understanding of the patients’ experiences with received information and possible distress in the diagnostic phase of PCa within both groups. The questions in the interview guide were designed to elaborate on the questions in the questionnaire, with three topics as focal points. These topics addressed consultations with the GP, consultations and communication with healthcare providers at the urological outpatient clinic, and possible distress (Table 1). At the beginning of each interview, the patients were encouraged to talk about their experiences from the time of the PSA test or Stockholm3 test until they received their biopsy result. The first author asked additional questions when elaboration was needed and only used the interview guide to ensure that all themes were discussed during the interview. The interviews lasted between 20 and 52 min.

**Table 1:** The semi-structured interview guide

**Regarding contact with the general practitioner (GP)**
What caused you to contact your GP? What information did you receive from your GP about the tests and blood tests that were performed? What information did you get from your GP if examinations were performed? What information did you get from your GP about how examinations would be performed? What information did you get from the GP of what would happen in the future? What information did you get from your GP about your consultation at the urological outpatient clinic? What information did you miss from your GP?
**Regarding contact with the urological outpatient clinic**
What information about your health condition have you received from a doctor/nurse at the urological outpatient clinic? What information did you receive on examinations and blood samples that have been performed, possibly scheduled for you? What information did you receive about examinations performed/possibly planned for you? What information did you receive about what should happen in the future? What information did you miss from the doctor/nurses at the urological outpatient clinic?
**Experienced anxiety and worries during the diagnostic phase of prostate cancer**
Why did you get a PSA^a^/Stockholm3 test? How did you experience the time until you received the result of the PSA/Stockholm3 blood test? How did you experience the time until the scheduled biopsy at the urological outpatient clinic? How did you experience the time until you received the biopsy result? What emotions arise when you think about prostate cancer? What do you think of the future?

^a^*PSA* Prostate-specific antigen

### Analysis

The quantitative data from the PSA group and the Stockholm3 group were statistically compared. The qualitative results for each of the two groups were subsequently compared before the results from both quantitative and qualitative analyses were merged.

#### Quantitative analysis

Initially, a power calculation was performed for an independent samples t-test and the required sample size was estimated to be 100 participants in each group to reach a power of 80% to detect a standardized difference of 0.4 with a two-sided significance level of 5%. The aim was increased to 120 in each group, to accommodate the ordinal nature of the outcomes and non-parametric testing [40].

The statistical analysis was performed using ordinal logistic regression. Descriptive methods were used to summarize the general characteristics of the participants and the distribution of the patient experience items using frequencies and percentages. Pearson’s chi-squared test was used to assess whether there was a statistically significant difference between the PSA group and the Stockholm3 group with regard to the categorical variables. Ordinal logistic regression was employed to compare the PSA group and the Stockholm3 group for each of the four dependent variables concerning patient experiences, while adjusting for age, living alone (yes/no) and education. The resulting odds ratios represent the relative odds of answering in a higher rather than lower category on the scales, in the Stockholm3 group vs. the PSA group. The proportional odds assumption was checked for all ordinal regression analyses using a likelihood ratio test, and all *p*-values were > 0.05. Binary logistic regression was conducted to compare proportions of potential anxiety and/or depression in the two groups. All odds ratios are presented with 95% confidence intervals and *p*-values from Wald tests. *P*-values ≤ 0.05 were considered statistically significant. Descriptive statistics and plots were produced using SPSS v. 26 and regression analysis was performed in Stata v. 16.1.

#### Qualitative analysis

The interview data were analyzed using Systematic Text Condensation (STC) [41]. STC consists of four steps: 1) Read the material to gain an overall impression and evoke preliminary themes, 2) Establish code groups (CG) from preliminary themes and identify meaning units that reflect the participant’s experiences of information, knowledge and distress, 3) Generate subgroups from the code groups and develop a condensate from the content in each subgroup and identify illustrating quotes, and finally 4) Synthesize condensates into conceptual descriptions [41]. The interview data from the two groups were analyzed separately.

Based on the notion that analysis benefits from collaboration, the full transcripts were distributed among the research team in order to create a wider analytic space [41]. After the team read the transcripts, preliminary themes and code groups were negotiated during several rounds of discussion. Each round was entered in an analysis journal. Subsequently, the first author identified meaning units and developed sub-categories, and categories, and after further negotiation in research team meetings, the final descriptions were presented to the research team. See Table 2 for a selected part of the analysis.

**Table 2:** Selected parts of the qualitative analysis

**Preliminary themes**	**Meaning units**	**Code groups** (CG)	**Condensate**	**Themes**
Fluctuating quality and quantity of information during the diagnostic phase of PCa	But it was in relation to the fact that the GP could have been a little more [forthcoming] before, uh, before, uh, before we started to get the results. Like what they mean and such, for the only thing he said was "We will send a referral", and I did not ask very much either, because actually I did not really know exactly what I should ask about (Stockholm3, ST247)	The level of information about the PSA, the Stockholm3 test and PCa depends on the individual healthcare providers (CG1)	He told me it was a special blood test that was sent to Stockholm. My GP told me it was a more accurate and safe test, compared to the PSA test, so I was happy … I received my Stockholm3 test result in a letter, it was slightly elevated, but what does that mean, could it be serious? My GP just called me and Informed me that he would refer me for further examinations (Stockholm3)	Information affects the experience of comprehension
	I wondered a little about being called in [to have a CT scan], you know, I did not get, you know, the invitation letter, with contrast fluid. It was sent by post and it had the wrong address because they did not have the same system as the Ward here had (Stockholm3, ST301) Yes, they have been very accessible. Very good and, as I said, very informative. They have been approachable and very friendly all of them [health care providers] (Stockholm3, ST207)	Predictability and adequate information are important for men's satisfaction and sense of security (CG2)	At first, I was told that they would not do any examinations at the hospital, then suddenly I received an appointment for a MRI, it was a little contradictory. Then they scheduled a biopsy as if suddenly there was an opening it came a little abruptly. There was a glitch in the system it was as if the order did not quite add up (Stockholm3) The Healthcare providers have been very welcoming and nice. They have explained things to me so that I was well prepared before examinations and not left in ignorance. I feel that they have taken care of me in a very professional manner (Stockholm3)	Stepping into the world of the healthcare system
Periodic distress and potential anxiety	In my youth, cancer… cancer was of course synonymous with death then. Now I am quite old, but when I was in my younger years, then there was no help or assistance or drugs. Cancer cures were not very successful at that time. If you got cancer, then as a rule you died. So some of that stays in my old mind, even though I know that things are going the opposite way today with most forms of cancer (PSA^a^, ID40)	Men’s experience of distress varies individually. (CG3)	I was very anxious before my first appointment at the urological outpatient clinic. It was not just because of the suspected PCa but rather a fear of pain and the unknown. Thoughts about PCa never left my consciousness, but I was never that worried (PSA^a^)	Periodically feelings of distress

#### Comparison

After STC was performed separately for each participant group, the final step of comparing themes and identifying key similarities and differences between the two groups was conducted. These findings were based on relevance, prevalence, and perceived importance of the data [42]. Themes and descriptions from both groups were compared systematically and constantly balanced against aim and context. Matrixes were used to organize descriptions of themes in order to establish an overview while identifying similarities and differences [43]. After comparing themes, the similarities and differences were discussed within the research team and the description was further adjusted according to the aim of the study until consensus was reached.

#### Rigor

Comparison between groups in qualitative research can add rigor and transferability to findings and facilitate the identification of key ingredients in the change that makes a difference [43]. Establishing confidence in qualitative data depends on qualifications, experience, and reflexivity among the researchers [44]. The research team had a multidisciplinary composition, which strengthened the process of reflexivity during the analysis. The team consisted of two researchers in nursing science with extensive experience with qualitative research, a specialist nurse in a PhD fellowship, and an experienced former GP with quantitative research experience. All members of the research team had previous experience with patients affected by cancer. The first author had former experience as a cancer coordinator for bladder and kidney cancer in the urological department. This position was located on another floor, but involved some contact with staff in clinic I. This previous experience provided important insight into the diagnostic phase of PCa, but it was important for us to prioritize uncovering preconceptions throughout the whole process of inquiry.

#### Merging the results

The separate results from the quantitative analysis and the qualitative analysis are merged to form the discussion. This step includes identifying common concepts across the results to determine if the results from the two sets of analyses confirm, disconfirm, or expand on each other [31]. The first author did the preliminary merging of the results, and after several rounds of evaluation, an editing agreement was reached between all authors.

## Results

In general, the PSA group and the Stockholm3 group were similar with respect to demographic characteristics, with no statistically significant differences (Table 3).

**Table 3:** Demographic characteristics of the total survey sample and the individual distribution for each clinic

	**Prostate specific antigen (PSA)**								**Stockholm 3**		
	**All included**		Clinic I		Clinic II		Clinic III		**Clinic I**		
	**N**	*%*	N	%	N	%	N	%	N	%	P*
Included	**130**	**100**	47	*36.2*	37	*28.5*	46	*35.4*	**120**	**100.0**	
Age group	**130**		47		37		46		**117**^a^		0.189
41–50	**4**	***3.1***	0	*0.0*	2	*5.4 *	2	*4.3*	**1**	***0.9***	
51–60	**21**	***16.2***	7	*14.9*	6	*16.2*	8	*17.4*	**16**	***13.7***	
61–70	**66**	***50.8***	23	*48.9*	23	*62.2*	20	*43.5*	**61**	***52.1***	
71–80	**31**	***23.8***	16	*34.0*	6	*16.2*	9	*19.6*	**37**	***31.6***	
81–90	**8**	***6.2***	1	*2.1*	0	*0.0*	7	*15.2*	**2**	***1.7***	
Living alone	**130**		47			*37*		*46*	**118**^a^		0.546
Yes	**20**	***15.4***	8	*17.0*	5	*13.5*	7	*15.2*	**15**	***12.7***	
No	**110**	***84.6***	39	*83.0*	32	*86.5*	39	*84.8*	**103**	***87.3***	
Education (higher degree)	**117**^**a**^		40^a^		36^a^		41^a^		**110**^**a**^		0.446
Yes	**39**	***33.3***	21	*52.5*	10	*27.8*	10	*24.4*	**42**	***38.2***	
No	**78**	***66.7***	19	*47.5*	26	*72.2*	31	*75.6*	**68**	***61.8***	
Occupation	**130**		47		37		46		**118**^a^		0.534
Employed	**56**	***43.1***	18	*38.3*	21	*56.8*	17	*37.0*	**39**	***33.0***	
Domestic worker	**1**	***0.8***	0	*0.0*	1	*2.7*	0	*0.0*	**1**	***0.9***	
Disability pension	**3**	***2.3***	2	*4.3*	0	*0.0*	1	*2.2*	**3**	***2.5***	
Rehabilitation	**2**	***1.5***	1	*2.1*	0	*0.0*	1	*2.2*	**1**	***0.9***	
Retired	**63**	***48.5***	25	*53.2*	15	*40.5*	23	*50.0*	**72**	***61.0***	
Under education	**4**	***3.1***	0	*0.0*	0	*0.0*	4	*8.7*	**2**	***1.7***	
Other	**1**	***0.8***	1	*2.1*	0	*0.0*	0	*0.0*	**0**	***0.0***	

^*^Pearson’s chi-squared test

^a^Some data missing

Demographic characteristics of the qualitative interview sample are presented in Table 4.

**Table 4:** Demographic characteristics of the interview sample

	Prostate specific antigen (*n* = 10)	Stockholm3 (*n* = 10)
Age group		
41–50	0	1
51–60	1	2
61–70	6	4
71–80	3	3
People in the household		
1 person	3	3
2 persons	6	6
≥ 3 persons	1	1
Education (year after primary school)		
0–3	1	7
4–5	6	3
7–9	3	0
Occupation status		
Employed	4	6
Retired	5	4
On rehabilitation	1	0
Prostate cancer		
Yes	6	7
No	4	3

### Quantitative results

**Fig. 4:**
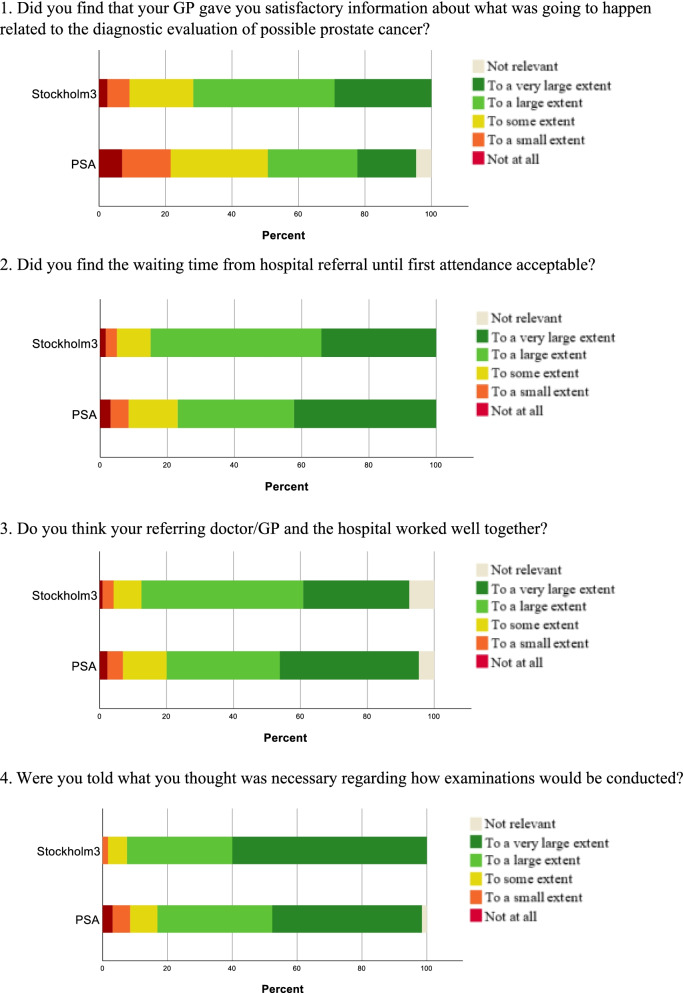
The percentage distribution of the responses in each of the four items

Figure 4 summarizes the frequencies in percentages of the total for each of the four items. For item 1, 53.2% in the PSA group and 28.3% in the Stockholm3 group responded “not at all”, “to a small extent”, or “to some extent” satisfactory. For item 2, item 3, and item 4 over 75% of the patients in both groups responded “to a large extent” or “to a very large extent”.

When comparing the two groups, the patients in the Stockholm3 group found the information provided by the GP more satisfactory than the PSA group (OR 2.61; 95% CI 1.59 to 4.28; *p* < 0.001) (item 1) (Table 5).

**Table 5:** Results on the patient experience survey and HADS

	Unadjusted				Adjusted^a^			
	N	*OR*	95% CI	*P*	N	*OR*	95% CI	*P*
Did you find that your GP gave you satisfactory information about what was going to happen related to the diagnostic evaluation of possible prostate cancer?	244	*2.45*	1.54—3.90	< 0.001*	221	*2.61*	1.59—4.28	< 0.001*
In your opinion, were you given the information you needed regarding the examination and how it would be done?	248	*1.87*	1.15—3.05	0.011*	224	*1.85*	1.10—3.11	0.020*
Did you find that the referring doctor/GP and the hospital cooperated well?	235	*0.90*	0.47—1.40	0.66	211	*0.83*	0.49—1.38	0.47
Did you find the waiting time from hospital referral until first attendance acceptable?	250	*0.95*	0.60—1.51	0.83	226	*0.50*	0.28—0.89	0.55
Anxiety (HADS^b^)	245	*1.11*	0.55—2.25	0.77	222	*1.05*	0.94—1.17	0.40
Depression (HADS^b^)	246	*1.79*	0.57—5.63	0.32	223	*1.87*	0.58—6.02	0.30
Anxiety and depression (HADS^b^)	246	*1.09*	0.44—2.73	0.85	223	*1.16*	0.45—2.98	0.76

^*^ Wald tests, *p*-values ≤ 0.05

^a^Adjusted for age, education and living alone (yes/no)

^b^HADS = Hospital Anxiety and Depression Scale

The Stockholm3 group also assessed the information they received regarding the further examination more sufficient (OR 1.85; 95% CI 1.10 to 3.11; *p* = 0.020) (item 4). No statistically significant differences were found regarding acceptance of waiting time from hospital referral until first attendance (item 2) (*p* = 0.55). Likewise, no statistically significant differences were found regarding how well the GP and the hospital worked together (item 3) (*p* = 0.47) (Table 5).

According to HADS, approximately 14% of the men in the PSA group and 15% of the men in the Stockholm3 group were at risk of developing anxiety (HADS-A). In the PSA group almost 4% of the men were at risk of developing depression, while almost 7% in the Stockholm3 group were at risk of developing symptoms of depression (HADS-D). About 8% were at risk of both (HADS-T) (Additional file 3). No statistically significant differences were found between the PSA group and the Stockholm3 group in terms of anxiety and depression (Table 5).

### Qualitative results

The STC analysis of the two groups resulted in three main themes with associated subthemes. The themes were systematically compared and the analysis reflected both differences and similarities between the two groups (see Table 6). The themes included “Information affects the experience of comprehension”, “Stepping into the world of the healthcare system”, and “Periodically feelings of distress”. For the theme “Periodically feelings of distress”, the analysis did not reveal any clear differences between the experiences of men in the Stockholm3 group compared to the PSA group. Accordingly, the results for this theme are presented together.

**Table 6:** Summary of thematic differences and similarities

Themes	Subthemes	Differences/ similarities	Prostate-specific antigen (PSA)	Stockholm3
1. Information affects the experience of comprehension	Initial introduction	Differences	Several patients reported that they had no initial information before the PSA test. Patients with several previous PSA tests often expressed that they had received sufficient information from their GP	All patientss reported that they had received some initial information before the Stockholm3 test
	Men’s perception of the diagnostic test	Differences	Patients explained that an elevated PSA was not always to be trusted. Besides prostate cancer, an elevated PSA could be a sign of infection or an enlarged prostate gland	In general, patients perceived the Stockholm3 test as a more accurate test. It was described as a medical progress
	Receiving the test result	Differences	For some patients, the elevated PSA level came as a shock because they were unaware of the test in the first place	Patients were aware of the Stockholm3 test and were prepared to receive the test result
		Similarities	The information patients received varied from being informed about a referral for further diagnostic evaluation to more comprehensive information	
2. Stepping into the world of the healthcare system	Trying to keep track of the diagnostic process	Differences	Patients were more inclined to report errors and delayed responses from the hospital	Overall, men were satisfied with the communication with the hospital. However, they did report some errors and delayed responses Some men believed that the fast and well organized diagnostic process was because they had agreed to the Stockholm3 test
		Similarities	In general, patients described the diagnostic phase of prostate cancer as well organized and fast, without too much unnecessary waiting. Not all patients identified the fast process as a part of the standardized care pathway, which made some patients worry about being seriously ill	
	Receiving information and care at the urological outpatient clinic	Similarities	Most patients described the healthcare providers as professional and caring. However, patients had very different needs for information; some required more detailed written and oral information both before and after the biopsy. The healthcare providers did not always identify these needs	
3. Periodically feelings of distress		Similarities	Patients did not experience pervasive anxiety. However, most men described times with worries or anxiety. The word “cancer” was associated with death. For some, anxiety became more prominent when the results of the biopsy approached. Others described both physical and psychological discomfort. It seemed that some patients found it difficult to explain or identify their different emotions during the diagnostic phase of prostate cancer	

### Theme 1: Information affects the experience of comprehension

The theme describes how patients in both groups experienced the information they received from their GP. The quality and amount of information affected how patients were prepared for the diagnostic process. The theme encompasses three subthemes: initial introduction, a more accurate test, and receiving the test result.

#### Initial introduction

The two groups described considerable differences in how the PSA and the Stockholm3 test were introduced to them. Most patients in the PSA group reported that they had received little or no initial information about PSA or the potential implications of a PSA test. A participant explained:

‘He [the GP] took a blood sample, uh I had no idea of what he was going to do with it’ (PSA, ID30).

For some patients, the lack of information led to bewilderment and frustration. In contrast, other patients indicated that they respected the GP’s decision about the PSA test. Regardless of the initial amount of information, the decision was interpreted as an expression of the GP’s care and professionalism. Upon further reflection, the GP’s choice of not including them in the decision about the PSA test was suggested as a natural consequence of the GP- patient relationship. Patients who had knowledge of elevated PSA over time reported repeated consultations with their GP where the subject had been discussed and that they had received sufficient information about PSA.

The Stockholm3 group said that they had received initial information about the Stockholm3 test and identified it as a diagnostic test. They expressed satisfaction with receiving initial information, which appeared to be considered a natural part of the consultation. Despite patients being aware of the test, it often seemed more like a recommendation than the GP providing sufficient information that enabled patients to make an informed decision.

#### Men’s perception of the diagnostic test

The comparison between the PSA group and the Stockholm3 group revealed very different perceptions of the diagnostic blood test that had been conducted. The main difference between the two groups was that the Stockholm3 group frequently characterized the Stockholm3 test as a more accurate test than the PSA test. This perception appeared to be initiated by the information provided by the GPs, who described the test as an extended and more trustworthy test. This is illustrated by the following patient’s response:

‘I guess I received information that they would do, well, a Stockholm test instead of the PSA because the Stockholm test seemed to be more accurate. So, if there was something there, they could catch it, and if there was nothing, then you avoided going through the whole process of setting off a huge mechanism [further diagnostic evaluation] and all that stuff. So, that's what I got from my GP’ (Stockholm3, ST207).

#### Receiving the test result

For patients in the PSA group who did not have knowledge of the test being performed, the elevated PSA test result came as a shock. Meanwhile, all patients in the Stockholm3 group expected to receive the test result and had considered the possibility of it being elevated. The amount of information patients received when their test results were presented differed within both groups. The information varied between a short message about the referral to the urological outpatient clinic to more thorough conversations about the test result. A patient remembered that his GP announced the Stockholm3 test result without further explanation:

‘No, nothing else except that he would refer me to this place [the hospital] so they could take a biopsy, quite simply’ (Stockholm3, ST267).

Another participant stated that he received information about potential side-effects of treatment simultaneously with the test result, and also explained that his GP had told him about the risk of overdiagnosis:

‘Yes, it might be that you, I would almost say, would be sexually incapacitated and you could get [urine] leakage and some such unpleasantness that they did not want you to get, so he [the GP] explained that’ (PSA, ID40).

In both groups, variations in the quality of information provided resulted in varying dispositions before entering the diagnostic phase of PCa. Nevertheless, the initial awareness of the Stockholm3 test seemed to generate a more comprehensible and clarified situation compared to the PSA group, which one average expressed more uncertainty about their elevated PSA test result.

### Theme 2: Stepping into the world of the healthcare system

This theme refers to how patients experience the encounter with the healthcare system. Two subthemes emerged: 1) trying to keep track of the diagnostic process and 2) receiving information and care at the urological outpatient clinic.

#### Trying to keep track of the diagnostic process

In general, patients in both groups appeared impressed or grateful to the Norwegian healthcare system, which they experienced as taking responsibility when it really mattered. However, patients in the PSA group seemed more inclined to report delayed or mixed up responses and consultations from the urological outpatient clinic. Patients in both groups referred to this as system errors in the communication pathway. The diagnostic phase of PCa was mostly considered to be well planned and without too much waiting time. Patients in both groups seemed somewhat puzzled because they did not know about the rapid diagnostic investigation, which could cause a discrepancy between the desire for diagnosis clarification and worries about a serious illness. They expressed that they were lucky when consultations were scheduled fast or they had received cancelled consultations. Furthermore, the patients had speculations about the fact that cancer was a serious illness and had to be given priority over less serious illnesses. In contrast to the PSA group, some men in the Stockholm3 group believed that their Stockholm3 test implied that the diagnostic process was accelerated:

‘Well, so actually it is quite impressive that, uh, uh, I don't know, so it is based on me being involved in research [Stockholm3] or something like that. I come to the doctor and get information: You will be examined and first there is the MRI [Magnetic resonance imaging], it takes no more than 14 days, actually. And it didn't. Then I went to have an MRI and the next day, then I got a phone call from the hospital, can you come and have an ultrasound today, quarter past one? Nothing further, but in fact am I seriously ill? What is it that makes that I, uh, that it happens so fast?’ (Stockholm3, ST226).

Some patients in both groups recognized the rapid diagnostic process as a part of the SCP.

#### Receiving information and care at the urological outpatient clinic

Patients identified consultations with the urologists as important time points for gaining information, whereas nurses had a more flexible role with more contact points in terms of disseminating personalized information. For example, these contact points could be conversations by phone with the coordinating nurse or receiving information from a nurse before and after the biopsy. The nurses could also mediate additional contact between the patients and the urologist. The healthcare providers were generally described as helpful, friendly, and professional. More specifically, the urologists were referred to as proficient in their job and the nurses as caring and skilled in conveying information. These competencies were perceived as important to ensure a safe and trusting environment for patients. A patient from the PSA group stated:

‘I feel that I am being extremely well looked after by the people [healthcare providers] who organise this and I think that inspires confidence and so I can relax and think: “Yeah, yeah, they know what they are doing and know their job” ’ (PSA, ID38).

The PSA and the Stockholm3 group received written information from the hospital that was sent by mail, as well as oral information from a nurse prior to the biopsy. The perception and need for written information varied within patients in both groups. Patients considered the information to be short and precise without causing unnecessary anxiety and implied that they did not have a need for detailed information. Some even deliberately did not pay attention to the written information, while others just skimmed through it:

‘Yes, I remember, I believe I read [the information], I believe that I probably read it through, so browsed it and then read a little ... (sighs), but I remember nothing now (chuckles)’ (PSA, ID36).

The reason for not reading the information carefully was that both groups believed that the healthcare providers told them what they needed to know regarding the biopsy. In contrast, some patients in both groups requested more detailed information and appeared less satisfied with the written information, which they believed failed to prepare them for what to expect during and after the biopsy. They also requested more oral information, especially about bowel leakage during the biopsy and prolonged bleeding after the biopsy. Patients in both groups requested more detailed information about sensitive issues, for example, blood in their semen.

### Theme 3: Periodically feelings of distress

This theme relates to the emotions and distress that could arise during the diagnostic phase of PCa. The strength of different emotions seemed to vary between patients and depend on individual assumptions or knowledge about PCa. There were no differences in emotional reactions between the two test groups, but rather some variations within the whole group of patients, regardless of test type.

The cancer diagnosis confronted patients with an immediate possibility of death, which could manifest in episodes of fear of death or catastrophic thoughts. A patient spontaneously declared:

‘Well, it is straight to the little white box [casket], isn't it? To me, cancer means death, you know, but of course it is not. So, “off the bat”, what cancer means to me, it means “Game Over”. I mean, doesn't it?’ (PSA, ID36).

Episodes of distress emerged in different situations, sometimes in solitude at night.

‘Clearly, when you have gone to bed a short while before the wife comes up [to bed], then you have thought: “Goodness, what if it is the beginning of the end, like?” ’ (Stockholm3, ST233).

Regardless of periodic feelings of distress, it was a common perception that the diagnostic phase of PCa was not associated with anxiety, but rather was something underlying that could not be entirely ignored. Patients expressed that they had felt anxious right before receiving the biopsy result. It seemed that the emotions experienced in the diagnostic phase could be difficult to identify or separate and therefore also difficult to explain.

## Discussion

In the present study, the quantitative and the qualitative results are merged and presented in a narrative discussion that organizes the quantitative and qualitative results side by side within a section of text [28]. The quantitative results showed that men in the Stockholm3 group were more likely to find the information from the GP more sufficient than men in the PSA group. These findings are supported by the qualitative results.

The Stockholm3 group was more than twice as likely (OR = 2.61) as the PSA group to find the information from the GP sufficient. During the interviews, several men in the PSA group explained that they had received little or no initial information about the PSA test. In contrast, patients from the Stockholm3 group had received initial information about the Stockholm3 test. Surprisingly, both groups seemed to find the GP’s behaviour natural, which may indicate that the patients trusted their GP’s judgement and his/her authority regardless of the initial information. The interaction between patients and doctors has previously been identified as complex and influenced by professional authority, which should be recognized during consultations [45]. Despite individual differences in information needs, none of the patients in the present study indicated having received too much information. This suggests that the information from healthcare providers was typically valued and appreciated and did not cause unnecessary distress.

Patients in the Stocholm3 group seemed more inclined to have confidence in the accuracy of their diagnostic test than men in the PSA group. This might suggest that the mandatory patient questions included in the Stockholm 3 algorithm supports more dialogue between the patient and the GP before the test is conducted. This is an important finding as previous research has shown that many patients are tested without having received adequate information or having made a shared decision as recommended [46]. A review found that public controversies regarding the PSA test caused some patients to feel confused and uncertain about the accuracy and reliability of the PSA test [47]. Kannan et al. 2019 reported that men in general did not fully understand what a PSA test entailed and some men were not familiar with the term PSA [19]. Another study found that less than 30% of patients received sufficient information about the accuracy of PSA and the risks and benefits of different PCa treatments [48].

There were no statistically significant differences between the groups regarding acceptance of waiting time from hospital referral until first attendance. This corroborates the qualitative results for the same theme. Errors that could cause delay and confusion were reported in both groups, while rapid diagnostic evaluations also made patients wonder about its urgency. This was in line with findings in a previous study, which found patients with suspected cancer may associate rapid diagnostic evaluation with individual diagnosis and prognosis and not as a part of the SCP, which possibly increased their worries [49].

The Stockholm3 group were more likely than the PSA group to state that they had received sufficient information regarding examinations when attending the outpatient clinic (OR = 1.85 [CI 1.10, 3.11], *p* < 0.05). However, the qualitative findings revealed no differences between the two groups. Patients in both groups expressed varied needs for information, from wishing as little information as possible to requesting detailed information. Wade et al. 2015 [50] found that when information about side-effects and sequelae differed from the actual experience, men tended to get more anxious and frustrated with the pre-biopsy information. Distress has been reported to be common after a prostate biopsy and is possibly caused by multiple factors, including the experience of the procedure, waiting for the result, uncertainty, and aspects of personality [27]. In the present study, the patients explained that nurses could be reached outside scheduled consultations and could thereby provide additional and more personalized information. Nurses also facilitated contact or information between the urologists and the patients if needed. Access to information has been identified as central for patients in order to manage uncertainty throughout the PCa pathway and in their evaluation of care. Good quality of care for PCa patients involves sufficient information, recognition of patients’ feelings, and effective and timely communication between the different healthcare providers [51].

Our study found no statistically significant differences between the two groups in terms of anxiety and depression. Approximately 14% of the patients in the PSA and 15% in the Stockholm3 group, who were awaiting a prostate biopsy at the outpatient clinic, experienced anxiety. Patients in both groups reported very similar experiences and did not appear to experience any significant anxiety, but periodic distress was common. Our findings emphasize that patients’ emotions and possible distress in the diagnostic phase are idiosyncratic, complex, of an inconsistent nature, and independent of the diagnostic test performed. The measurement of anxiety may be complicated further as patients may not define their emotions as anxiety or worry but still experience psychological distress that affects their wellbeing. Correspondingly, a review found that anxiety in patients affected by PCa appeared to vary over the clinical timeline [52]. Another study reported that 41% of men awaiting a biopsy result showed some degree of distress with anxiety being more dominant in those men [53]. Patients in the diagnostic phase of PCa are a group at risk of psychological distress, and some patients may benefit from additional support and information [24, 54]. Nurses and doctors have an important role to play in identifying patients affected by distress and providing tailored information and support.

### Strengths and limitations

In this study, trailing research was found to be appropriate to study patients’ experiences with the diagnostic phase of PCa. Furthermore, the design enables the use of different methods [29]. The intent of the convergent mixed methods design is that the quantitative and qualitative methods complement each other [28]. In this study, the qualitative results elaborated on the quantitative results. Therefore, the conclusions that are presented may be considered more robust than they otherwise would have been should only one analytic approach have been used in isolation [55]. Ideally, all data should have been collected from Clinic I, but due to the rapid introduction of the Stockholm3 test, two more clinics were added to complete the data collection. Although the three clinics had similar procedures according to the SCP and adhered to the same health trust, local differences must be expected, which could have affected the results at each clinic. In addition, the novelty of the Stockholm3 test may have affected the information provided by the GPs. Another limitation is that we have no report of how many patients declined to participate in the study. The results should be considered in the context of this limitation. In future research, one should attempt to repeat the data collection in a single clinic and in a larger scale.

When using comparison groups, homogeneity of demographics characteristics should be pursued as much as limitations allow [43]. The two groups differed in age and education, but were otherwise very similar. The composition of the two groups may have affected the findings. Since the comparison groups explore similarities and differences within a particular context and in the nature of qualitative research, the results may not be generalizable. In qualitative research, the researcher might influence the interview unconsciously, but the research team tried to avoid biases by uncovering preconceptions both before and after the interviews. Despite limitations of the study, the quantitative and qualitative results complemented each other, which enhanced the overall validity of the study.

## Conclusion

We found that men who had a Stocholm3 test had received more sufficient information from their GP compared to men who had a PSA test. Therefore, patients in the Stockholm3 group felt more prepared when they received the result of their diagnostic test. However, information about potential risks and benefits regarding diagnostic testing and side-effects of treatment for PCa seemed insufficient in both groups. The Stockholm3 test may facilitate the provision of information to patients; however, further research is needed to explore ways to enhance the amount of information received prior to and after a Stockholm3 test. In both groups, nurses were identified as a source of additional and more personalized information. Nurses have a critical position for providing additional support and mediate contact between urologists and patients. Routines that ensure more sufficient information should be a priority in order to provide patients with greater predictability and to avoid unnecessary distress. Patients at risk of psychological distress or anxiety may experience particular benefits from early detection and initiated actions.

## Supplementary Information

**Additional file 1.** Good Reporting of A Mixed Methods Study (GRAMMS*) Checklist.

**Additional file 2.** Counts and percentages of patients with different response categories to the four patient experience items in the PSA^a^group (*n*=130) and the Stockholm3 group (*n*=120).

**Additional file 3.** Counts and percentages of patients with scores above threshold levels for the Hospital Anxiety and Depression Scale in the PSA^a^group (*n*=130) and the Stockholm3 group (*n*=120).

## Data Availability

The dataset analysed during the current study is available from the corresponding author on reasonable request.

## Abbreviations

PSA: Prostate specific antigen
GRAMMS: Guidelines for reporting mixed-methods research
PCa: Prostate cancer
ERQCC: Essential Requirements for Quality Cancer Care
SCP: Standardized patient cancer pathway
GP: General practitioner
HADS: The Hospital Anxiety and Depression Scale
STC: Systematic Text Condensation
CG: Code group
