# Supplementary material for: Men’s perception of information and psychological distress in the diagnostic phase of prostate cancer: a comparative mixed methods study
Source: BMC Nurs. 2022 Sep 30;21:266. doi: 10.1186/s12912-022-01047-1 (PMC9526317; doi:10.1186/s12912-022-01047-1)
Supplement: Supplementary file 1 — Additional file 1. Good Reporting of A Mixed Methods Study (GRAMMS*) Checklist. [file 12912_2022_1047_MOESM1_ESM.docx]

**Additional File 1** Good Reporting of A Mixed Methods Study (GRAMMS*) Checklist

| Guideline | Section: page |
| --- | --- |
| Describe the justification for using a mixed methods approach to the research question | Methods - under Design pp 8-9 |
| Describe the design in terms of the purpose, priority and sequence of methods | Methods - under Design pp 7-9 |
| Describe each method in terms of sampling, data collection and analysis | Methods - under  Setting and sample pp 10-11  Data collection p 11  Quantitative measurement pp 11-12  Qualitative interviews p 13  Analysis pp 14-18 |
| Describe where integration has occurred, how it has occurred and who has participated in it | Methods - under  Merging the results p 18  Discussion p 31 |
| Describe any limitation of one method associated with the present of the other method | Discussion – under  Strengths and limitations pp 34-35 |
| Describe any insights gained from mixing or integrating methods | Discussion – under  Strengths and limitations pp 34-35 |

* O'Cathain A, Murphy E, Nicholl J. The quality of mixed methods studies in health services research. Journal of Health Services Research and Policy. 2008;13: 92-98
