# Supplementary material for: Men’s perception of information and psychological distress in the diagnostic phase of prostate cancer: a comparative mixed methods study
Source: BMC Nurs. 2022 Sep 30;21:266. doi: 10.1186/s12912-022-01047-1 (PMC9526317; doi:10.1186/s12912-022-01047-1)
Supplement: Supplementary file 2 — Additional file 2. Counts and percentages of patients with different response categories to the four patient experience items in the PSAagroup (n=130) and the Stockholm3 group (n=120). [file 12912_2022_1047_MOESM2_ESM.docx]

**Additional file 2** Counts and percentages of patients with different response categories to the four patient experience items
in the PSA^a^ group (n=130) and the Stockholm3 group (n=120)

| Question |  | 1. Did you find that your GP gave you satisfactory information about what was going to happen related to the diagnostic evaluation of possible prostate cancer? | | | |  | 2. Did you find the waiting time from hospital referral until first attendance acceptable? | | | |  | 3. Did you find that the referring doctor/GP and the hospital worked well together? | | | |  | 4. Were you told what you thought was necessary regarding how examinations would be conducted? | | | |
| --- | --- | --- | --- | --- | --- | --- | --- | --- | --- | --- | --- | --- | --- | --- | --- | --- | --- | --- | --- | --- |
| Response |  | **PSA**  N=130 | | **Stockholm3**  N=120 | |  | **PSA**  N=130 | | **Stockholm3**  N=120 | |  | **PSA**  N=130 | | **Stockholm3**  N=117^b^ | |  | **PSA**  N=129^b^ | | **Stockholm3**  N=120 | |
|  |  | N | *%* | N | *%* |  | N | *%* | N | *%* |  | N | *%* | N | *%* |  | N | % | N | % |
| Not at all |  | 9 | *7.3* | 3 | *2.5* |  | 4 | *3.1* | 2 | *1.7* |  | 3 | *2.4* | 1 | *0.9* |  | 4 | *3.1* | 0 | *0* |
| To a small extent |  | 19 | *15.3* | 8 | *6.6* |  | 7 | *5.4* | 4 | *3.3* |  | 6 | *4.8* | 3 | *2.7* |  | 7 | *5.5* | 2 | *1.7* |
| To some extent |  | 38 | *30.6* | 23 | *19.2* |  | 19 | *14.6* | 12 | *10.0* |  | 17 | *13.7* | 10 | *9.1* |  | 11 | *8.6* | 7 | *5.8* |
| To a large extent |  | 35 | *28.2* | 51 | *42.5* |  | 45 | *34.6* | 61 | *50.8* |  | 44 | *35.5* | 58 | *52.7* |  | 46 | *35.9* | 39 | *32.5* |
| To a very large extent |  | 23 | *18.6* | 35 | *29.2* |  | 55 | *42.3* | 41 | *34.2* |  | 54 | *43.6* | 38 | *34.6* |  | 60 | *46.9* | 72 | *60.0* |
| Not applicable |  | 6^c^ |  | 0 |  |  | 0 |  | 0 |  |  | 6^c^ |  | 7^c^ |  |  | 1^c^ |  | 0 |  |

a PSA = Prostate-specific antigen

b Missing data

c Not applicable left out of the calculation of percentages
