# Supplementary material for: Men’s perception of information and psychological distress in the diagnostic phase of prostate cancer: a comparative mixed methods study
Source: BMC Nurs. 2022 Sep 30;21:266. doi: 10.1186/s12912-022-01047-1 (PMC9526317; doi:10.1186/s12912-022-01047-1)
Supplement: Supplementary file 3 — Additional file 3. Counts and percentages of patients with scores above threshold levels for the Hospital Anxiety and Depression Scale in the PSAagroup (n=130) and the Stockholm3 group (n=120). [file 12912_2022_1047_MOESM3_ESM.docx]

**ADDITIONAL FILE 3** Counts and percentages of patients with scores above threshold levels for the Hospital Anxiety and depression Scale
in the PSA^a^ group (n=130) and the Stockholm3 group (n=120)

| HADS^b^ score |  |  | **PSA group** | | |  |  | **Stockholm3 group** | | ***P**** |
| --- | --- | --- | --- | --- | --- | --- | --- | --- | --- | --- |
|  |  |  |  | | |  |  |  | |  |
|  |  | n_total_ | n | *%* |  | | n_total_ | n | *%* |  |
| ≥8 Anxiety (HADS^b^-A) |  | 128 | 18 | *14.1* | |  | 117 | 18 | *15.4* | 0.770 |
| ≥8 Depression (HADS^b^-D) |  | 128 | 5 | *3.9* | |  | 118 | 8 | *6.8* | 0.314 |
| ≥15 Anxiety and depression (HADS^b^-T) |  | 128 | 10 | *7.8* | |  | 118 | 17 | *8.5* | 0.849 |

* Pearson’s chi-squared test

a PSA = Prostate-specific antigen

b HADS = Hospital Anxiety and Depression Scale

c Two participants in the PSA group and three participants in the Stockholm3 group had missing data
